# P22 Exploration of specialist AMS nursing roles across the UK and Ireland

**DOI:** 10.1093/jacamr/dlad143.026

**Published:** 2024-01-03

**Authors:** Emma Mewse, Sue Bowler, Jo McEwen, Enrique Castro-Sánchez

**Affiliations:** Guy’s and St Thomas’ NHS Foundation Trust, London, UK; Nottingham University Hospital NHS Foundation Trust, UK; Ninewells Hospital and Medical School, Dundee, UK; Brunel University London, Uxbridge, UK

## Abstract

**Background:**

There is increasing interest in the work undertaken by nurses employed as antimicrobial stewardship (AMS) specialists across the UK and Ireland but scarce evidence describing their roles, characteristics and career trajectory is available.

**Objectives:**

To investigate the characteristics of the current specialist AMS nursing workforce in the UK and Ireland to inform progression and support. Specifically, to (i) describe the background, roles and professional development opportunities of specialist AMS nurses; and (ii) identify barriers and enablers in delivering the specialist AMS nursing role.

**Methods:**

An online survey was circulated among purposefully selected members of the AMS Nursing Group and Scottish Antimicrobial Nursing Group (SANG). Survey development was informed by the literature^1^ and included closed questions (*n*=23) alongside free text, with expert validation of content and access. Consent was assumed on survey completion, responses were anonymized.

**Results:**

Twelve registered and one non-registered nurse completed the survey (100% completion rate), AfC band 5 to 8 or equivalent, from England (*n*=8), Scotland (*n*=3), Wales (*n*=1) and Ireland (*n*=1). All respondents were directly involved in clinical audit, education and quality improvement projects (*n*=13), alongside contributing to IV to oral switch initiatives (*n*=12), AMS ward rounds (*n*=9) and the clinical management of infection (*n*=7). Most respondents capture the impact of these interventions with process measures, clinical outcomes or feedback (*n*=11). Respondents work closely with microbiology/infectious diseases teams (*n*=13), infection prevention and control (*n*=12), ward teams (*n*=11) and pharmacy colleagues (*n*=9). Ambition to progress in current roles was noted, including development as clinical academic, nurse consultant/ANP or within regional or national teams (*n*=6). Opportunity to access further formal qualifications to support AMS roles including degree, masters or postgraduate modules can be limited (*n*=5). Free text responses revealed both barriers and facilitating factors to delivering the specialist AMS nursing role. Challenges included dual role commitments, being part of a pilot project and lack of understanding of the nursing role in AMS. Enablers included support from multi-professional colleagues and autonomous working.

**Conclusions:**

AMS programmes are currently being supported by specialist AMS nurses. This survey revealed that there are commonalties and variations, as well as barriers and challenges to delivering the role. Interprofessional collaborative working and learning was evident, reflecting the multi-professional nature of AMS. These roles are therefore in a position to influence nursing clinical practice and behaviours^2^ in collaboration with other specialist teams.^3^ Opportunities exist to formalize the AMS nursing role and ensure inclusion as a core member of the AMS team within UK and Ireland. This should be part of an integral strategy to support the largest workforce within the NHS and requires recognition and commitment at team, trust and national level.
